# Molecular farming navigates a complex regulatory landscape

**DOI:** 10.3389/fpls.2024.1411943

**Published:** 2024-07-16

**Authors:** Jack Bobo

**Affiliations:** Faculty of Science, Food Systems Institute, University of Nottingham, Nottingham, United Kingdom

**Keywords:** molecular farming, genetic engineering, alternative proteins, biotechnology, regulations, religion, ethics

## Abstract

Molecular farming, the practice of engineering plants to produce recombinant proteins, presents novel challenges and opportunities for domestic markets and international trade. This article explores the multifaceted risks associated with these biotechnological advancements, including public health concerns related to recombinant animal proteins produced in plants, cross-contamination and unintended allergens, and the necessity for stringent identity preservation systems to avoid past failures. On the global stage, the trade of such genetically engineered crops brings about unique regulatory concerns, underscoring the need for internationally harmonized policies and reevaluating existing low-level presence (LLP) thresholds to address unexpected allergens. Moreover, molecular farming ventures into complex religious and ethical territories, particularly affecting communities with strict dietary laws, such as Islamic, Jewish, and those following vegan or vegetarian lifestyles. Addressing these concerns requires a collaborative approach among scientists, regulatory bodies, industry leaders, and religious figures, aiming to foster an inclusive dialogue that navigates the ethical, religious, and environmental implications of integrating animal proteins into plant-based systems. Such efforts are essential for ensuring the responsible development of molecular farming technologies, contributing to a future of sustainable, secure, and inclusive food systems that respect diverse cultural and ethical values.

## 1 Introduction

As the global population grows from 8 billion people today to as many as 9.5 billion by the year 2050, the quest to sustainably and nutritiously feed an expanding population while limiting environmental harm becomes ever more challenging. The 20th century saw significant advancements in agricultural productivity, resulting in substantial declines in global hunger. However, this came with considerable ecological impact, including growing land degradation, water scarcity, deforestation, and greenhouse gas emissions.

In the face of these challenges, many companies are exploring innovative methods to produce essential nutrients with a smaller environmental footprint. Plant-based proteins and precision fermentation technologies are well-advanced. Other technologies, like cultivated meat made from animal cells in bioreactors, have only recently received regulatory approval in some countries and have yet to be proven at scale.

Some companies are exploring using genetic engineering to modify plants to produce animal proteins. This process, known as molecular farming, involves genetically modifying plants to synthesize specific substances, including animal proteins (Long et al., 2022). These engineered plants are processed to extract the targeted substance and channeled into the appropriate commercial supply chain (Dietz and Muldoon-Jacobs, 2024). Although not a new concept, this strategy has recently gained renewed interest for its potential to manufacture valuable proteins on a large scale. However, the potential of molecular farming of animal proteins comes with significant regulatory considerations.

This article explores the food safety, trade, and religious/ethical implications of molecular farming of plants that produce animal proteins, as well as considerations for their stewardship to protect public health and religious or ethical requirements or preferences.

## 2 Risky business

The advent of molecular farming of animal proteins in plants has clear implications for food safety. Some animal proteins under consideration for molecular farming are known allergens. The introduction of allergens into food plants—where such allergens have historically been absent—poses unique challenges. Molecular farming could lead to allergenic animal proteins being unexpectedly present in foods, catching allergic consumers off guard, and raising public safety concerns.

The idea of transferring known allergens into plants is contrary to international standards developed by Codex Alimentarius (Codex) on the regulation of genetically engineered (GE) plants. Codex is an international food safety standard setting body with more than 180 member countries. In 2003 Codex established principles and guidelines for the safety assessment of foods derived from genetic engineering to provide the basis for allergenicity risk assessment strategies by regulators worldwide (EFSA Panel on Genetically Modified Organisms (GMO) et al., 2022). Codex guidelines discourage the transfer of genes from commonly allergenic food, stating, “The transfer of genes from commonly allergenic foods and from foods known to elicit gluten-sensitive enteropathy in sensitive individuals should be avoided unless it is documented that the transferred gene does not code for an allergen or for a protein involved in gluten-sensitive enteropathy.” (Codex Alimentarius Commission, 2003) By contrast, molecular farming products of animal proteins in plants involves the intentional transfer of genes from allergenic sources to food crops, including animal genes known to code for allergenic proteins. As a result, molecular farming of allergenic animal proteins in plants presents difficult questions for regulators.

A familiar cautionary tale for GE developers of genetically modified plants is a case from the 1990s where a Brazil nut gene was transferred to soybeans to increase protein content. The 2S albumin from Brazil nut expressed in experimental transgenic soybean was recognized by IgE antibodies from sera of patients allergic to Brazil nut, suggesting that this protein was a Brazil nut allergen and that it was a probable allergen too if expressed in soybean. The research was immediately terminated (Nordlee et al., 1996).

In response to these emerging concerns, the FDA proactively issued guidance in April 2023, outlining critical considerations for developers engaging in the transference of allergens into novel food crops (Food and Drug Administration, 2023) (Food and Drug Administration, 2023). The risks associated with food allergens are not to be underestimated, as even trace amounts can trigger severe, potentially life-threatening allergic reactions. The primary defense for individuals with food allergies remains to avoid known allergens, typically managed through careful label reading on packaged foods.

For instance, a consumer with a milk protein allergy habitually checks food labels to avoid milk products. However, the hypothetical scenario of food plants engineered to produce milk proteins mixed with conventional food products complicates this straightforward practice. Such commingling could render a wide range of soy-based products unsuspected vectors for the milk allergen, depriving consumers of the necessary information to avoid allergens effectively. The regulatory hurdles have not deterred product developers from developing genetically engineered plants to produce the dairy protein casein, a known allergen. This risk may result in heightened regulatory oversight and agricultural practices in cultivating, processing, and labeling to safeguard those with food allergies and the integrity of the supply chain. (Dietz and Muldoon-Jacobs, 2024)

## 3 New challenges for identity preservation systems

The deployment of molecular farming for food crops containing animal proteins and novel allergens requires careful oversight to prevent these novel proteins from inadvertently contaminating the food supply. The unintended presence of these proteins has public health and ethical implications for consumers with allergies as well as those with religious and ethical dietary restrictions.

Crops engineered to contain novel traits are not new. Nor are management systems (also known as identity preservation or closed-loop systems) to keep these products segregated from the rest of the food supply. Technology producers, farmers, and grain handlers have successfully established identity preservation systems to ensure that quality traits can be maintained separate from the traditional commodity system to meet product specifications. Identity preservation systems are closed-loop systems intended to ensure that crops containing specific quality traits, such as organic foods and oilseeds with modified oil profiles, are not mixed with varieties that do not contain the quality trait. This is usually done to maintain a product quality, which may result in a product premium. Importantly, existing identity preservation systems are not present to keep crops with quality traits out of the general food supply.

Such identity preservation systems have successfully handled genetically engineered crops with specific quality improvements, like altered fatty acid composition in oilseeds. However, their potential effectiveness in managing crops modified to contain allergens—particularly in staple foods—is unclear. The strategies used to maintain certain quality traits do not necessarily apply to the challenges of preventing allergenic risks. Quality management aims to preserve the intended characteristics of a crop. Ensuring safety from allergens (and religious and ethical requirements) requires more rigorous measures. This is because the stakes are higher with allergens. A failure to manage these risks could lead to severe health consequences for allergic individuals beyond compromising the crop’s quality or intended traits. (Dietz and Muldoon-Jacobs, 2024)

## 4 Past failures

Experience has shown that total segregation of food crops intended for different purposes is challenging, even with extensive identity preservations systems and stewardship programs of the crops in the field. Previous failures in identity preservation have often resulted from human error or the failure of specific management protocols (APHIS, 2020). The StarLink corn incident represents a critical juncture in U.S. regulatory bodies’ oversight of genetically engineered products. StarLink was designed to express the Bacillus thuringiensis (Bt) protein, Cry9C, a pesticidal protein that conferred resistance to certain pests, making it a significant agricultural innovation. However, due to concerns about its potential allergenicity—a risk that had not been conclusively ruled out—the U.S. Environmental Protection Agency (EPA) granted Starlink corn regulatory approval exclusively for use in animal feed, not for human food (Cry9C protein from Starlink corn: contains a single mutation that renders it resistant to gastro-intestinal digestion, see the original EPA assessment of Starlink, section A.3 and the summary of study 442581-8 in Table 1 mentioning the lack of digestibility; Bucchini and Goldman, 2002).

Dietz and Muldoon-Jacobs discuss the repercussions of the failure to segregate the product, which was never determined to pose a public health risk, in their article *Molecular farming- importance of stewarding food crops engineered to produce transferred food allergens and non-food substances*, “Data available to EPA indicate that 4 million tests were performed on 4 billion bushels of corn. StarLink corn was planted on about 600,000 acres over the course of 3 years which represented less than half a percent of the total acreage planted to corn in the United States.” (Dietz and Muldoon-Jacobs, 2024)

Given past stewardship failures, FDA’s 2023 guidance on molecular farming urged developers to consider their ability to ensure total segregation in the product development and production process. The guidance stated, “We believe it is critically important to consider whether you and your partners throughout the supply chain can reliably establish and maintain conditions, from farm to processing to consumption, under which such new plant varieties, and protein-containing materials from such varieties, do not inadvertently enter the food supply, and are properly labeled when they are intentionally part of the food supply.” (Food and Drug Administration, 2023)

In the early stages of developing products produced through molecular farming of animal proteins in plants, developers must rigorously evaluate the capability to securely manage these crops to prevent them from unintentionally entering the food supply. FDA’s guidance explains that this entails adopting comprehensive management strategies covering the entire production and distribution process, including seed production, planting, harvesting, storage, transport, processing, and formulation, to mitigate risks such as inadvertent commingling. This level of stewardship is critical to protect consumers by ensuring the safety of the food supply. (Dietz and Muldoon-Jacobs, 2024) Producing crops in contained facilities such as greenhouses or vertical farms would reduce the risk of commingling compared to production in open fields. The selection of a production host will also impact the complexity of maintaining segregation.

### 4.1 Trade disruptions

The previous section highlighted the complications that would arise if potential food allergens from molecular farming were to appear in domestic foods. The appearance of such products in trade could lead to similar regulatory concerns in importing countries even if the exporting country determined that no recalls were necessary.

The international trade of genetically engineered crops has significantly developed since their introduction in the 1990s. Key crops such as soybeans, corn, cotton, and canola have dominated the GE crop market, accounting for a substantial portion of the international trade volume of these crops, with key growing regions in North and South America. Herbicide-tolerant and insect-resistant varieties have become the most widely adopted trait types (Clarke et al., 2013).While technology developers have endeavored to seek approvals for new GE varieties in import markets, there have been numerous examples of trade disruptions resulting from asynchronous approvals, where products are approved and marketed in exporting countries before approvals in importing countries are finalized (Mendoza Cuello et al., 2020). The advent of molecular farming of animal proteins in food crops presents a fundamentally different risk to trade compared with previously commercialized GE crops.

Trade problems arise when technology developers in exporting countries commercialize approved products before foreign approval is complete. This can occur because the product developer believes foreign approval will occur before the crop is harvested and enters international trade or because they intend to keep the product out of global trade. The low-level presence (LLP) of unapproved products may result in trade disruptions.

The detection of unapproved products in trade has resulted in numerous trade disruptions both in terms of delays due to trade being blocked as well as in terms of the financial impact of the trade disruptions. According to the Food and Agriculture Organization, 74 countries had reported LLP incidents involving GE products as of 2014. (Mendoza Cuello et al., 2020) In November 2007, the Codex Alimentarius Commission *Ad Hoc* Intergovernmental Task Force on Foods Derived from Biotechnology produced an annex to the Codex plant guideline that addresses safety assessments in situations of low-level presence (LLP) of GE plant material in commodity shipments and food products. The guidelines relate to situations where GE products have completed a food safety review in at least one country (Langridge et al., 2014). Many countries have now implemented LLP policies to reduce the risk of trade disruptions should low levels of unapproved GE products appear in trade. For example, in 2015, Japan established a 5 percent threshold for unapproved GE products in commodity shipments (Gruere, 2009; Sato, 2016).

Without LLP policies, shipments have traditionally been deflected to countries where products have been approved. This might be appropriate if animal proteins are detected in animal feed. However, such an approach would not work for human food as regulators are unlikely to allow even small amounts of allergens in the food supply where the presence is unknown to the consumer.

## 5 Religious and ethical considerations

The advent of molecular farming, which can produce animal proteins in plant hosts, introduces novel and complex questions to the regulation of these products that lies at the intersection of science, ethics, and religious dietary law. Molecular farming’s potential to integrate genes from non-permissible sources, such as pigs, into widely consumed plant products may prompt reevaluating traditional nutritional practices and beliefs, particularly within communities adhering to strict dietary guidelines, such as those prescribed by Islamic and Jewish laws, as well as vegan and vegetarian diets.

For Muslim communities, the concept of halal, which is integral to Islamic dietary laws, mandates that food be permissible, wholesome, and beneficial. In Malaysia, the Institut Kefahaman Islam Malaysia and the Jabatan Kemajuan Islam Malaysia have determined that GE foods are halal, provided they originate from halal sources and are produced through halal methods. However, introducing genes from non-halal sources, such as pigs, into plants raises significant religious and ethical concerns. The acceptability of GE products largely depends on the source of the donor gene. For example, genetically engineered chymosin, which replaces animal-derived rennet in cheese production, has been widely accepted as halal, provided the manufacturing process adheres to Islamic principles. By contrast, the halal status of genetically engineered foods containing animal genes, particularly those resembling porcine genes, has not yet been addressed (Qureshi, 2017).

The distinction between synthetic genes created through recombinant technology and actual animal material in molecular farming may be crucial to some communities. Even if analogous to a porcine gene, a synthetic gene may be permissible in halal GE products through molecular farming, provided it contains no actual porcine material. This nuanced approach underscores the importance of thorough review and evaluation by religious authorities to ensure compliance with halal dietary laws, reflecting the complex interplay between science, ethics, and religion in genetically modified food production. (Qureshi, 2017)

While Islamic scholars grapple with the nuances of halal certification for these novel foods, Jewish dietary law appears more accommodating of genetic engineering, emphasizing the non-specific nature of DNA and the permissibility of genetic modifications that do not involve direct crossbreeding. Interestingly, rabbinic scholars have already explored the question of introducing animal genes in plants to produce animal proteins. “Most rabbinic scholars agree that genetic engineering does not violate the divine order, and that biblical verses prohibiting mating ‘diverse kinds’ apply only to true mating and therefore are inapplicable to new technologies such as genetic engineering. With this understanding, there is no need for concern about the possibility of a gene from a non-kosher food item being transferred to one that is kosher; in fact, because all DNA is made of the same basic material, it is misleading to refer to a pig gene as uniquely porcine.” (Glasgow, 2015) While it may be too early to declare such products acceptable to Jewish consumers, it does suggest that the question may not be quite as complicated as that facing Islamic scholars.

The ethical dilemma may extend beyond the Muslim community. Producing animal proteins in food crops may raise concerns for individuals maintaining vegan or vegetarian diets. Veganism and vegetarianism are moral positions that oppose exploiting and otherwise harming nonhuman animals. While many scholars speak to ethical questions about what foods are or are not vegan or vegetarian, there are no equivalents to the Islamic and rabbinic bodies that can establish guidelines for the entire community (Deckers, 2016). As a result, there will likely be a broader range of positions on whether animal proteins produced in plants are acceptable. The case of cultivated meat demonstrates the range of perspectives. While there is generally wide agreement that cultivated meat products are meat and, therefore, not technically vegan or vegetarian. However, in one informal survey, nearly 25% of vegans reported being comfortable consuming these products (Pointing, 2023). As a result, vegan and vegetarian consumers are likely to be even more willing to consume animal proteins produced through molecular farming.

The intersection of molecular farming with religious and ethical considerations presents a complex landscape that challenges traditional dietary laws and ethical stances. While molecular farming offers the potential to produce animal proteins in plants, thereby introducing genes from non-permissible sources into widely consumed foods, it raises significant concerns within communities adhering to Islamic and Jewish dietary laws, as well as among individuals following vegan or vegetarian lifestyles. The acceptability of such genetically engineered (GE) products hinges on nuanced interpretations of religious laws and ethical principles, necessitating thorough review and evaluation by religious authorities and ethical scholars. Islamic dietary laws, for instance, require a careful examination of the source of genes and the production process to determine the halal status of GE foods, whereas Jewish dietary laws show a more accommodating stance towards genetic engineering, highlighting the non-specific nature of DNA. Similarly, the acceptance of these novel foods among vegan and vegetarian communities may vary, reflecting diverse ethical considerations and personal choices. As molecular farming continues to evolve, ongoing dialogue among religious scholars, ethicists, and the broader community will be crucial in navigating the ethical and religious implications of these biotechnological advancements, ensuring that the development and consumption of GE foods align with diverse cultural, religious, and ethical values.

## 6 Discussion

Molecular farming of animal proteins in plants for domestic markets and trade raises new risks. In addition to the public health risk these new proteins may pose and the heightened stewardship protocols required, the products also present novel religious and ethical questions. As we navigate the intricate landscape of molecular farming, it becomes clear that collaboration among scientists, regulators, industry stakeholders, and religious leaders is crucial. By fostering an inclusive dialogue that addresses the multifaceted implications of introducing animal proteins into plant-based systems, we can pave the way for the responsible advancement of molecular farming technologies. This collective effort will be instrumental in realizing the potential of molecular farming to contribute to a sustainable, secure, and inclusive food future, aligning technological progress with ethical, religious, and environmental considerations.

## Data availability statement

The original contributions presented in the study are included in the article/supplementary material. Further inquiries can be directed to the corresponding author.

## Author contributions

JB: Writing – original draft, Writing – review & editing.
